# Transcriptome analysis of parallel-evolved *Escherichia coli *strains under ethanol stress

**DOI:** 10.1186/1471-2164-11-579

**Published:** 2010-10-19

**Authors:** Takaaki Horinouchi, Kuniyasu Tamaoka, Chikara Furusawa, Naoaki Ono, Shingo Suzuki, Takashi Hirasawa, Tetsuya Yomo, Hiroshi Shimizu

**Affiliations:** 1 Department of Bioinformatic Engineering, Graduate School of Information Science and Technology, Osaka University, 1-5 Yamadaoka, Suita, Osaka, Japan; 2 Exploratory Research for Advanced Technology (ERATO), Japan Science and Technology Agency (JST), 1-5 Yamadaoka, Suita, Osaka, Japan; 3 Graduate School of Frontier Biosciences, Osaka University, 1-5 Yamadaoka, Suita, Osaka, Japan

## Abstract

**Background:**

Understanding ethanol tolerance in microorganisms is important for the improvement of bioethanol production. Hence, we performed parallel-evolution experiments using *Escherichia coli *cells under ethanol stress to determine the phenotypic changes necessary for ethanol tolerance.

**Results:**

After cultivation of 1,000 generations under 5% ethanol stress, we obtained 6 ethanol-tolerant strains that showed an approximately 2-fold increase in their specific growth rate in comparison with their ancestor. Expression analysis using microarrays revealed that common expression changes occurred during the adaptive evolution to the ethanol stress environment. Biosynthetic pathways of amino acids, including tryptophan, histidine, and branched-chain amino acids, were commonly up-regulated in the tolerant strains, suggesting that activating these pathways is involved in the development of ethanol tolerance. In support of this hypothesis, supplementation of isoleucine, tryptophan, and histidine to the culture medium increased the specific growth rate under ethanol stress. Furthermore, genes related to iron ion metabolism were commonly up-regulated in the tolerant strains, which suggests the change in intracellular redox state during adaptive evolution.

**Conclusions:**

The common phenotypic changes in the ethanol-tolerant strains we identified could provide a fundamental basis for designing ethanol-tolerant strains for industrial purposes.

## Background

Experimental evolution is a powerful tool for the study of the evolution of emergent properties in biological systems. This experimental system enables us to clarify phenotypic and genotypic changes responsible for adaptive evolution [1,2]. Parallel-evolution experiments can be performed under identical conditions, and they enable us to distinguish which phenotypic and genotypic changes are inevitable for adaptive evolution and which occurred by mere chance. For engineering purposes, the outcomes of such evolution experiments have the potential to provide valuable information for the rational design of useful strains [3]. For example, by long-term cultivation of a microorganism under an environmental stress conditions, we can expect to obtain stress-tolerant strains after cycles of mutation and selection. The mechanisms of stress tolerance can be elucidated by analyzing the phenotype and genotype of the tolerant strains. By elucidating these mechanisms, we can develop strategies to induce this tolerance in other strains, such as industrially used strains. Screening of strains following random mutagenesis has been used to obtain strains with desired phenotypes [4-6]. However, the advantage of long-term experimental evolution in comparison with random mutagenesis and screening is that it enables the enrichment of beneficial phenotypic and genetic changes by iterative selections. Thus, the identification of essential factors for higher fitness is expected to be easier in experimental evolution.

In this study, we performed a series of evolution experiments to analyze ethanol tolerance in *Escherichia coli *cells. This microorganism is widely used in the production of useful materials, including amino acids, enzymes, biofuels, biopolymers, and others [7-9], and its importance in the production of biofuels from biomass resources has recently increased [10]. In the production of ethanol by this microorganism, ethanol is a major stress factor that interferes with growth and ethanol production. Thus, developing ethanol tolerance in *E. coli *strains is important for the improvement of ethanol production. In fact, the construction of ethanol tolerant strains of several microorganisms, such as *Saccharomyces cerevisiae*, were performed for the improvement of ethanol productivity, for example, by changing lipid composition of cell membrane and activation of amino acid biosynthesis pathways [11-13]. The screening and expression analysis of ethanol tolerant *E. coli *strain was also performed [14,15], which revealed the expression changes of several genes in the ethanol tolerant strain, such as increased metabolism of glycine and betaine, suggesting that these expression changes are involved in the mechanism of ethanol tolerance. However, in this previous study, the ethanol tolerance was analyzed by using a single clone of tolerant strain, and thus the mechanisms necessary for the ethanol tolerance is obscure. In this study, we analyzed several ethanol-tolerant strains obtained by an independent series of evolution experiments, which enabled us to identify common characteristics among the tolerant strains that should be involved in ethanol tolerance. We performed 6 independent series of evolution experiments under ethanol stress for over about 1,000 generations and obtained ethanol-tolerant strains that exhibited about 2-fold increase in specific growth rate compared to the parent strain. To understand the phenotypic changes in these strains, we performed comprehensive gene expression analysis of these tolerant strains by microarrays, and identified genes and functional categories with significantly up- or down-regulated expression among the tolerant strains. We found that genes involved in the iron ion transport and biosynthesis pathways of some amino acids, including tryptophan, histidine, valine, leucine, and isoleucine, were commonly up-regulated in tolerant strains, which suggests that these gene functions are involved in ethanol tolerance. In support of this hypothesis, we confirmed that the addition of isoleucine, tryptophan, and histidine to the culture medium increased the growth rate of the parent strain under ethanol stress. The comprehensive analysis of several ethanol-tolerant strains of *E. coli *provides clues to understanding the mechanism of ethanol tolerance.

## Results and discussion

### Parallel laboratory evolution experiments of *E. coli *under ethanol stress

**Figure 1:**
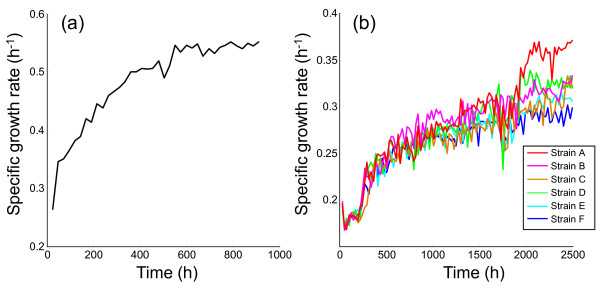
**Changes in specific growth rate during evolution experiments**. The time course of specific growth rates in the evolution experiments (a) without ethanol and (b) with 5% (v/v) ethanol are plotted. In the case with ethanol stress, 6 parallel series of experiments were performed starting from strain P obtained at 912 hours in the experiment shown in (a). The cells obtained after 2,500 hours of cultivation under ethanol stress were named "strain A"-"strain F," in descending order of the final growth rate.

Before starting the evolution experiments under ethanol stress, we performed a laboratory evolution experiment in M9 synthetic medium without the addition of ethanol to distinguish the phenotypic and genetic changes that occurred in the adaptive evolution to M9 synthetic medium from those that occurred due to ethanol stress. In Figure 1(a), we plot the change in specific growth rate during the evolution experiment without the addition of ethanol, which was carried out by serial transfer of an aliquot of culture to the fresh M9 medium every 24 hours starting from *E. coli *wild-type strain W3110. The specific growth rate increased by 2-fold and ceased to increase after approximately 1,000 hours of cultivation (700 generations). The *E. coli *population at 912 hours was stored at -80 °C and used as the parent strain in subsequent evolution experiments, which was named strain P throughout the paper. Six parallel-evolution experiments under 5% (v/v) ethanol stress were carried out starting from strain P in the same manner as in the experiments without ethanol addition. In these evolution experiments, we confirmed that the cells were maintained in the exponential growth phase and a considerable amount of glucose remained in the medium after a 24-hour cultivation. Figure 1(b) shows the change in specific growth rates in these evolution experiments under 5% ethanol stress. As shown in the figure, the specific growth rates gradually increased, resulting in an approximately 2-fold increase compared with strain P. We stored the evolved strains (named strains A-F in descending order of the final growth rate) at -80 °C and used them for further analysis of the phenotypic changes that occurred in the adaptive evolution to ethanol stress.

Table 1 shows the specific growth rates of the wild-type strain of *E. coli *(W3110), strain P obtained by the adaptive evolution to the M9 synthetic medium, and the evolved strains under ethanol stress (strains A and F) in M9 medium with varying ethanol concentrations (0, 5, 6, 6.5, and 7%). The data were obtained using frozen stocks of the strains, and the specific growth rates were calculated in the cultures with various ethanol concentrations after pre-culture with M9 medium without ethanol stress. As shown in Table 1, evolved strains A and F exhibited higher growth rates than the other strains, including in the 6.5% ethanol stress condition in which the wild-type strain W3110 and strain P cannot grow. In contrast, the growth rates of evolved strains were significantly lower than that of strain P in M9 medium that did not contain ethanol, suggesting the existence of evolutionary trade-offs in the adaptive evolution to ethanol stress, as reported in the case of adaptive evolution to different culture temperatures [16].

**Table 1:** Effect of ethanol concentration on the growth of each *E. coli *strain

Ethanol concentration (%)	**Specific growth rate (h**^**-1**^**)**			
	
	Wild type	Strain P	Strain A	Strain F
0	0.357 ± 0.006	0.584 ± 0.005	0.385 ± 0.008	0.510 ± 0.015
5	0.130 ± 0.005	0.168 ± 0.003	0.350 ± 0.008	0.288 ± 0.007
6	0.023 ± 0.009	0.058 ± 0.004	0.187 ± 0.006	0.177 ± 0.005
6.5	No growth	No growth	0.045 ± 0.006	0.009 ± 0.009
7	No growth	No growth	No growth	No growth

We confirmed that the phenotype of the evolved strains, i.e., higher growth rate under ethanol stress, was stable after cultivating them in M9 medium that did not contain ethanol for more than 100 generations (144 hours). After 100 generations in M9 medium that did not contain ethanol, the specific growth rate of tolerant strains A and F under 5% ethanol stress were 0.345 ± 0.020 and 0.315 ± 0.012 (h^-1^), respectively, which were similar to those observed after the adaptive evolution shown in Figure 1(b). Measurements by phase-contrast microscopy revealed that there is no significant morphological change (size, shape) between the wild-type strain W3110 and evolved strains (data not shown).

### Transcriptome analysis of evolved strains

**Figure 2:**
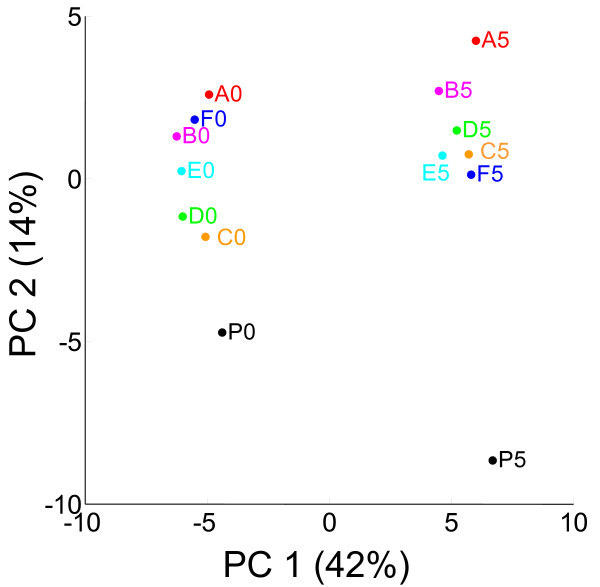
**PCA score plot of PC1 vs PC2**. P0 and A0-F0 represent the expression profiles of strain P and tolerant strains A-F obtained without ethanol addition, respectively. P5 and A5-F5 indicate the data for the 5% ethanol condition.

To analyze the phenotypic changes that occurred during adaptive evolution to ethanol stress, we performed microarray expression analysis of parent strain P and 6 tolerant strains, A-F, in M9 medium that contained and did not contain 5% ethanol (14 arrays used in total; complete data are presented in Additional file 1). For the microarray analysis, all strains were cultured in M9 medium without ethanol as a pre-culture. These strains were then cultured in the medium with and without ethanol for several generations to obtain samples in the mid-exponential phase. For the microarray data analysis, we used modified finite hybridize (FH) model to quantify absolute expression levels of genes, in which hybridization free energy between probes on the array and target DNA fragments is estimated from the signal intensities and probe sequences [17]. Since the number of expression profiles to be analyzed was large, we used principal component analysis (PCA) to represent the changes in the expression levels caused by both the addition of ethanol into the medium and adaptive evolution to ethanol stress. Figure 2 shows the result of PCA of these 14 expression profiles, in which principal components (PC) 1 and 2 explain 42 and 14% of the variance of expression data, respectively. As clearly shown in Figure 2, data points corresponding to the addition of ethanol (depicted as P5, A5-F5) are at the right while those corresponding to the absence of ethanol are at left, indicating that PC1 represents changes in expression levels in response to ethanol stress regardless of phenotypic changes in adaptive evolution. Furthermore, in both cases (i.e., with and without ethanol addition), the data points of strain P are located at the bottom of the figure, while those of the evolved strains are located at the top. It should also be noted that for the expression profiles obtained under the ethanol stress addition (P5, A5-F5), the order of data points along the PC2 axis roughly corresponds to growth rate under ethanol stress [see Figure 1(b)]. This result indicates that PC2 represents the change in expression levels that occurred during adaptive evolution to achieve higher growth rates under ethanol stress. That is, PCA of the microarray profiles was able to distinguish the expression changes in the strains occurred in the response to ethanol stress and in the adaptive evolution to the stress. Thus, to analyze the changes in expression levels in more details, we screened functional categories in which genes contributing to PC1 or PC2 were statistically overrepresented. Table 2 shows a list of functional categories in which genes having the top 5% strong positive or negative loadings for PC1 or PC2 are significantly overrepresented (p < 0.005; determined by the hypergeometric test). It should be noted that PCA and the subsequent statistical analysis were performed using expression data of only genes that were correctly quantified in all samples (2,317 of 4,499), and genes with low expression levels were excluded from the analysis to remove noisy data.

**Table 2:** Functional categories of genes that significantly contribute to PC1 and PC2.

Function	*P *value	Screened members	Total members	Gene names
**PC 1 top 5%**				
Galactitol metabolic process	5.98E-06	4	4	*gatB, gatC, gatY, gatZ*
Phosphate transport	3.58E-07	6	8	*phoB, phoU, pstA, pstB, pstC, pstS*
Phosphoenolpyruvate-dependent sugar phosphotransferase system	3.01E-04	6	20	*dhaH, gatB, gatC, manX, manY, manZ*
Response to stress	9.57E-06	13	64	*clpB, degP, dnaK, grpE, hslJ, hslU, htpG, ldhA, pstS, uspG, relB, relE, yfiA*
Response to heat	1.16E-05	8	24	*dnaK, groL, groS, grpE, hslJ, hslU, htpG, ldhA*
Protein folding	1.64E-05	8	25	*degP, dnaK, dsbA, groS, groL, grpE, htpG, ppiA*
**PC 1 bottom 5%**				
Cellular amino acid biosynthetic process	3.73E-08	20	100	*argF, argI, aroF, aroM, carA, carB, hisA, hisB, hisC, hisD, hisF, hisG, hisH, hisI, leuL, lysC, metH, pheA, thrL, trpL*
Histidine biosynthetic process	4.53E-09	8	11	*hisA, hisB, hisC, hisD, hisF, hisG, hisH, hisI*
Arginine biosynthetic process	3.00E-03	4	13	*argF, argI, carA, carB*
Tricarboxylic acid cycle	4.05E-04	6	21	*fumA, mqo, sdhA, sdhB, sdhC, sdhD*
Transport	1.21E-04	33	351	*amtB, argT, betT, emrA, entD, fadL, fiu, kgtP, livF, livG, livH, livJ, livK, livM, modA, modB, ompF, oppA, oppB, oppC, oppD, oppF, proP, putP, rbsB, rfbX, sdhA, sdhB, sdhC, sdhD, tsx, uraA, yhbE*
Amino acid transport	1.63E-03	8	46	*putP, argT, livF, livG, livM, livH, livK, livJ*,
Peptide transport	1.91E-03	5	19	*oppA, oppB, oppC, oppD, oppF*
Flagellum organization	4.72E-04	3	4	*flgC, flgD, flhM*
**PC 2 top 5%**				
Cellular amino acid biosynthetic process	1.49E-10	23	100	*argH, aroF, carB, hisB, hisC, hisD, hisH, ilvA, ilvB, ilvC, ilvD, ilvE, ilvM, leuA, leuB, leuC, leuD, trpB, trpC, trpD, trpE, trpL, tyrA*
Histidine biosynthetic process	1.50E-03	4	11	*hisB, hisC, hisD, hisH*
Tryptophan biosynthetic process	1.67E-06	5	6	*trpB, trpC, trpD, trpE, trpL*
Branched-chain family amino acid biosynthetic process	9.76E-10	10	17	*ilvA, ilvB, ilvC, ilvD, ilvE, ilvM, leuA, leuB, leuC, leuD*
Iron ion transport	5.47E-17	18	30	*cirA, entA, entB, entC, entD, entE, entF, fecA, fecB, fecI, fecR, fepA, fepB, fepC, fes, fhuE, fiu, mntH*
Enterobactin biosynthetic process	6.62E-10	7	7	*entA, entB, entC, entD, entE, entF, ybdB*
Iron-sulfur cluster assembly	1.05E-04	5	11	*hscA, iscS, sufA, sufB, sufD*
**PC 2 bottom 5%**				
Lipopolysaccharide biosynthetic process	2.70E-06	12	49	*eptB, kdtA, htrL, rfaB, rfaC, rfaF, rfaG, rfaI, rfaP, rfaQ, rfaS, rfaY*
Dipeptide transport	8.29E-05	4	6	*dppB, dppC, dppD, dppF*

Among genes with high loadings on PC1, which correspond to genes commonly up-regulated in response to ethanol stress, we found that genes related to the galactitol metabolic process are significantly overrepresented. In Fig. S1(a) presented in Additional file 2, we show the expression levels of *gat *genes with high loadings on PC1 (*gatB, gatC, gatY, gatZ*). As shown in the figure, these *gat *genes were commonly up-regulated in response to ethanol stress both for strain P and ethanol-tolerant strains. The *gat *genes are involved in biofilm formation [18] and are known to be up-regulated in response to several stresses, such as acid stress [19,20]. The genes related to phosphate transport (*phoB, phoU, pstB, pstA, pstC, pstS*) were also commonly up-regulated in response to ethanol stress [Fig. S1(b) in Additional file 2]. These genes are known to be regulated by the PhoR/PhoB 2-component regulatory system in response to change in extracellular phosphate concentration [21]. PhoR/PhoB system is also known to be involved in acid stress response [22]. In a previous study of isobutanol response network of *E. coli*, PhoB-regulated genes are up-regulated in response to isobutanol stress presumably due to the stress-induced disruption of quinone membrane interaction [23]. Our results suggest that a similar mechanism is involved in the response to ethanol stress. The *manXYZ *genes encoding subunits of phosphotransferase system for mannose uptake were also significantly up-regulated in response to ethanol stress for all strains [Fig. S1(c) presented in additonal file 2]. Okouchi *et al. *have shown that *manXYZ *genes are related to the response to solvent stress, such as *n*-hexane, cyclohexane, *p*-xylene, and toluene [24], and are highly up-regulated at both the transcript and protein levels under *n*-butanol stress [25]. Our results indicate that the changes in expression levels of *manXYZ *are also involved in the response to ethanol stress. Furthermore, we found that genes involved in the category "heat stress response" were significantly up-regulated in the response to ethanol stress, which includes genes encoding chaperon proteins (e.g., *groS*, *groL*, *grpE*, and *dnaK*). This result is consistent with that in previous studies, in which the heat-shock regulatory gene *rpoH *and its downstream genes are up-regulated when cells are exposed to ethanol [26], *n*-butanol [25], and isobutanol [23].

As for the genes with low loadings on PC1, which correspond to genes commonly down-regulated in response to ethanol stress, we found that genes related to histidine and arginine biosynthesis were significantly overrepresented, while the expression levels of genes in other pathways of amino acid biosynthesis were relatively unchanged. In Figs. S1(d) and (e), we show the expression levels of representative genes in these pathways. Although the mechanism for this down-regulation is unclear, our results might suggest that the inactivation of these pathways play a role in response to ethanol stress. Furthermore, we found that genes related to flagella biosynthesis were down-regulated in response to the addition of ethanol. Although most genes in this category were excluded from the statistical analysis shown in Table 2 due to their low expression levels in the presence of ethanol, we confirmed that almost all flagella-related genes were severely down-regulated in response to ethanol stress. We show the expression levels of some representative genes related to this category in Fig. S1(f). The decrease in the activity of flagella biosynthesis under ethanol stress was confirmed by using motility assay on soft agar plate (data not shown), as in the responses to other stresses such as heat stress and osmotic stress [27].

**Figure 3:**
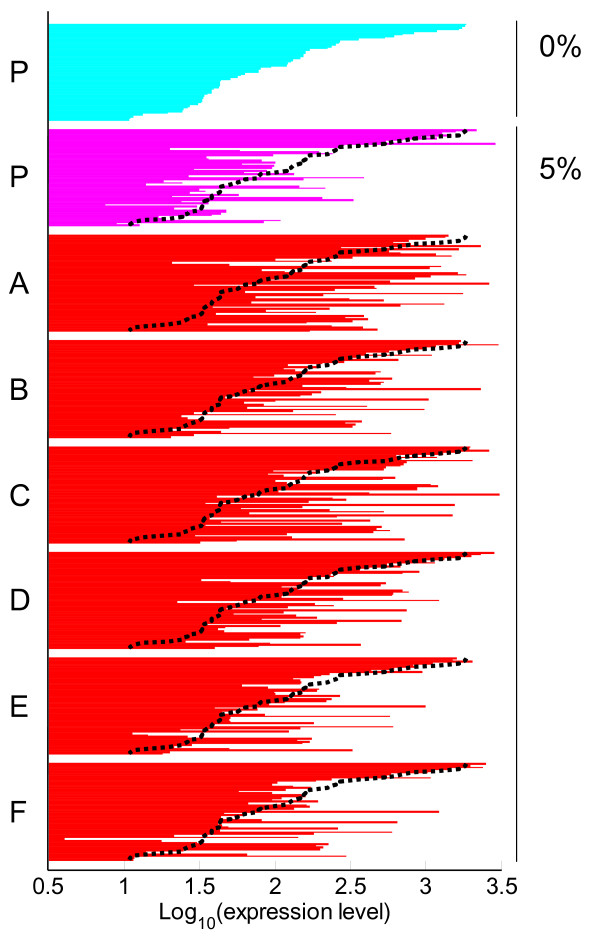
**Expression levels of genes regulated by Fur**. The log_10_-transformed expression levels of Fur regulon genes which were successfully quantified (57 genes in total) in strain P (0% or 5% ethanol) and in tolerant strains A-F (5%) are presented. The expression levels were sorted in the decreasing order of the expression levels in strain P without ethanol stress. Black dot lines represent the expression levels in strain P without ethanol stress for the reference. A list of Fur regulon member genes in the same order is presented in Additional file 4.

In the result of PCA shown in Figure 2, PC2 represents the changes in expression levels that occurred during the adaptive evolution to ethanol stress. On this component, we found that genes related to iron ion metabolism ("iron ion transport", "enterobactin biosynthetic process", and "iron-sulfur cluster assembly") had significantly high loading factors, indicating that these genes were up-regulated during the adaptive evolution to ethanol stress. It is well known that Fur, a global regulator of iron ion transport, represses the transcription of these iron ion transport-related genes [28]. To confirm the possibility that adaptive evolution to ethanol resulted in the change of Fur repressor function, we plot the expression levels of genes which are known to be repressed by Fur in strain P and tolerant strains A-F (Figure 3). As shown in the figure, Fur regulon members generally exhibited higher expression levels in the tolerant strains than in strain P. One possible explanation for the activation of iron ion metabolism genes is that the enhancement of iron ion uptake is involved in the ethanol stress tolerance. Another possible explanation is that it is caused by the change in intracellular redox state. Brynildsen and Liao reported that Fur regulon genes in *E. coli *are generally down-regulated in response to isobutanol addition to the culture medium, and this down-regulation was suggested to be due to the decrease in intracellular superoxide ion (${\text{O}}_{2}^{-}$ ) concentration caused by the quinone/quinol malfunction [23]. It was also shown that the increase of both intracellular and hydrogen peroxide levels, which invoke the oxidative stress response, increases the expression levels of iron-import genes through the inactivation of Fur [29,30]. From these previous studies and our data, we hypothesized that during the adaptive evolution to the ethanol stress environment, the cells changed their respiratory system, which resulted to increase in intracellular ${\text{O}}_{2}^{-}$ or hydrogen peroxide level. This hypothesis was supported by the fact that genes regulated by OxyR and NrdR were commonly up-regulated in the tolerant strains, as shown in Fig. S2 presented in Additional file 3, which are known to respond to oxidative stress [31,32]. The detailed mechanisms for the change in redox state are unclear, and further investigation should be performed to clarify the relationship between the adaptation to ethanol and the change in the respiratory system. Furthermore, as for the genes with high loadings on PC2, we found that the genes related to the biosynthesis of tryptophan, histidine, and branched-chain amino acids (valine, leucine, and isoleucine) were significantly up-regulated in the tolerant strains, as shown in Fig. S1(g), (d), and (h) presented in Additional file 2, suggesting that the activation of these biosynthetic pathways in involved in the development of ethanol tolerance. There are several reports about the relationship between the activation of several amino acids biosynthesis or amino acid supplementation and environmental stress tolerance. For example, extracellular glutamate and arginine are involved in acid stress resistance, and glycine and proline are known to be osmoprotectant [33]. However, the relationship between activation of amino acids biosynthetic pathways we found (i.e., tryptophan, histidine, valine, leucine, isoleucine) and tolerance to ethanol and other stresses in *E. coli *was not reported so far. In yeast *S. cerevisiae*, it was reported that yeast strains overexpressing tryptophan biosynthesis genes showed ethanol stress tolerance [13], and the supplementation of isoleucine, methionine, and phenylalanine into the medium resulted ethanol stress tolerance [34]. Furthermore, the fact that intra-cellular amino acids concentrations increase under several environmental stress conditions, including cold, heat, and oxidative stresses, suggested that the concentration increase of amino acids within cells contribute to tolerance to these stresses in *E. coli *[35]. Our data suggested that also in *E. coli*, the activation of some amino acids biosynthetic pathways contribute to ethanol stress tolerance.

We also found that genes related to lipopolysaccharide (LPS) biosynthesis were generally down-regulated in the tolerant strains in comparison with strain P, except for tolerant strain C. Fig. S1(i) also shows the expression levels of some representative genes of this pathway. LPS is associated with permeability to hydrophobic molecules and is related to defense against stress [36]. The inactivation of LPS biosynthesis might suggest that a change in the outer membrane occurred during adaptive evolution to the ethanol stress environment. It was reported that the increased levels of unsaturated fatty acids are important for ethanol tolerance in *Saccharomyces cerevisiae *and *E. coli *[37,38]. However, in the tolerant strains we obtained, there are no significant changes in the expression levels of genes related to fatty acid biosynthesis in comparison with the strain P.

In addition to the expression changes common to all tolerant strains as discussed above, there were expression changes specifically occurred in each tolerant strain. For example, under ethanol stress condition, genes involved in the methionine biosynthesis pathway were significantly down-regulated in strain A and strain C in comparison with other strains [Fig. S1(j)]. Note that, strain A and strain C exhibited relatively higher growth rates under ethanol than the other tolerant strains except for strain B, and thus the down-regulation of methionine related genes in these two strains might be responsible for their higher growth rates. The development of ethanol tolerance by the down-regulation of methionine related genes might be possible when increasing production of some metabolites which share the same precursor with methionine, such as those derived from oxaloacetate, is responsible for the ethanol tolerance. Such analysis of specific expression changes in each tolerant strain might be helpful to illustrate the mechanisms of ethanol tolerance in more details.

### Effect of amino acids and iron ion supplementation on ethanol tolerance of *E. coli*

**Figure 4:**
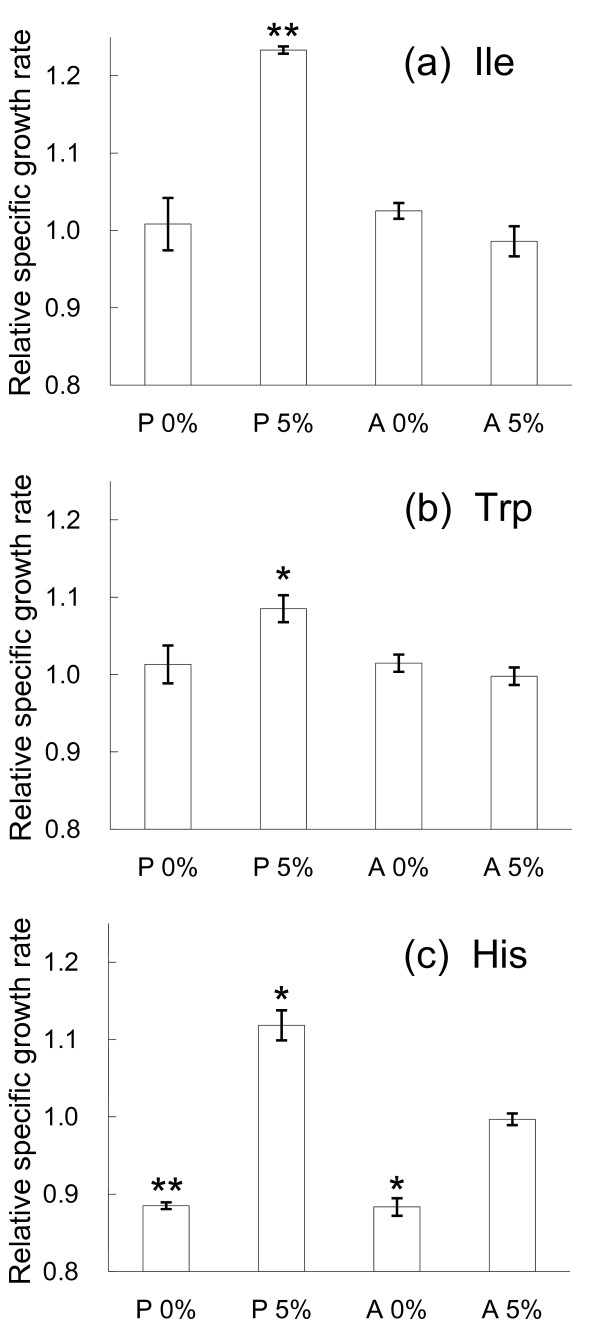
**Change in specific growth rates through supplementation of amino acids**. The relative specific growth rate, i.e., specific growth rate in the presence the amino acid supplementation divided by that in the absence of the supplementation, are presented. (a) isoleucine supplementation in a final concentration of 0.1 mM; (b) tryptophan and (c) histidine supplementation in a final concentration of 1 mM are plotted for the cases of strain P and A with and without the ethanol stress addition. The error bars represent the standard deviation from 3 replicate measurements. An asterisk indicates a *P*-value of < 0.02, two asterisks indicate a *P*-value of < 0.002, respectively, determined by *t*-test.

From the results of microarray expression analysis, it was speculated that *E. coli *cells can acquire ethanol stress tolerance through the enhancement of the expression levels of genes related to amino acid biosynthetic pathways (tryptophan, histidine, valine, leucine, and isoleucine) and iron ion transport machinery. To further investigate this possibility, we evaluated the effect of amino acid and iron ion supplementation into the medium on the growth in the presence or absence of ethanol stress. We tested the effect of tryptophan, histidine, valine, leucine, and isoleucine supplementation into the media, respectively, in a final concentration range from 0.1 mM to 10 mM. In Figure 4, we plot the fold increase in the specific growth rate, i.e., the specific growth rate in the presence the amino acid supplementation divided by that in the absence of the supplementation, which exhibited significant growth changes under the ethanol stress. As shown in the Figure 4(a), the specific growth rate of strain P cultivated in M9 medium with 5% ethanol and 0.1 mM isoleucine was significantly higher than that in the medium without isoleucine supplementation, while the increase of growth rate was not observed in the absence of ethanol stress. A similar increase of the specific growth rate was observed from 1 mM tryptophan supplementation [Figure 4(b)]. The supplementation of histidine resulted in the significant decrease in the specific growth rate in the case without ethanol stress addition, while the growth enhancement was observed only in the case of strain P with addition of the ethanol stress [Figure 4(c)]. The results suggested that the increase of intra-cellular concentrations of these amino acids were a part of the mechanism for the ethanol tolerance achieved by the experimental evolution. In contrast, for the tolerant strain A, these amino acids supplementation exhibited no growth enhancement both in the conditions with and without ethanol stress addition. This might be due to that the up-regulation of these amino acids biosynthesis related genes in the tolerant strain A is enough to enrich the intra-cellular concentration of the amino acids required for the ethanol stress tolerance. The increase in specific growth rate through amino acid supplementation is much smaller than that observed in the long-term culture experiments shown in Figure 1(b), which might suggested that the activation of these amino acids biosynthetic pathways is a part of stress tolerant machinery and there are other mechanisms involved in the observed ethanol tolerance. Supplementation of other amino acids and iron ion had no effect on the specific growth rate under ethanol stress (data not shown).

## Conclusion

In this study, a series of evolution experiments was performed to investigate the adaptive evolution of *E. coli *under conditions of ethanol stress. We obtained 6 ethanol-tolerant strains through independent long-term culture experiments. These strains showed an approximately 2-fold increase in specific growth rate under 5% ethanol stress. Comprehensive gene expression analysis of the tolerant strains revealed that common changes in expression levels occurred among the tolerant strains we obtained, which strongly suggests that these phenotypic changes are involved in the development of ethanol tolerance. We found that genes related to iron ion metabolism were commonly up-regulated in the tolerant strains, which suggests that a change in the redox state occurs during adaptive evolution. We also found that the genes related to biosynthetic pathways of tryptophan, histidine, valine, leucine, and isoleucine were commonly up-regulated in the tolerant strains. The activation of these amino acid biosynthesis pathways is speculated to be responsible for the ethanol stress tolerance we observed, and this hypothesis was partially supported by the finding that supplementation of isoleucine, tryptophan, and histidine into the medium increases the specific growth rate under an ethanol stress environment. These findings should be a starting point of understanding the molecular mechanisms involved in the ethanol stress tolerance in *E. coli*, and thus can be fundamental knowledge for designing ethanol-tolerant *E. coli *cells for the improvement of ethanol productivity in the industry.

The common expression changes observed in ethanol tolerant strains A-F showed little overlap with previous studies about ethanol tolerance in *E. coli *cells [15,39]. Among the common expression changes in the tolerant strains we identified, only the up-regulations of enterobactin biosynthesis genes, which are involved in iron ion metabolism, were already reported as those related to ethanol tolerance [39]. In Ref. [39], genes and their functions related to ethanol tolerance in *E. coli *were screened by using a comprehensive transposon mutant library and an overexpression library. In this study, in addition to enterobactin biosynthesis, genes related to osmoregulation and cell-wall biogenesis were found to be involved in the ethanol tolerance. In another previous study about ethanol tolerance in *E. coli *[15], ethanol-tolerant strains were obtained by using serial transfer culture experiments. Microarray expression analysis of the ethanol tolerant strain revealed that genes regulated by FNR, which mediates the transition from aerobic to anaerobic growth, were significantly down-regulated and aromatic amino acid biosynthesis (*aroF*, *aroG*, *aroL*, and *tyrA*) and glycine metabolism (*gcvT*, *gcvP*, and *lpdA*) are up-regulated in comparison with the control strain. In our data, the expression changes of genes in these previously screened categories were not observed except for those related to enterobactin. These differences can be due to the differences in the methodology for the screening and the environmental condition used for the experiments. For example, in the previous studies Luria-Bertani medium was used for the cultivation of *E. coli *cells, while we used the synthetic medium without amino acids.

Whole-genome resequencing analysis of the tolerant strains will provide information on the mutations that caused the observed phenotypic changes during adaptive evolution. Our preliminary results of whole-genome resequencing analysis showed that there were little overlaps among identified mutations in the tolerant strains we obtained, indicating that no cross-contamination occurred during the parallel-evolution experiments (data not shown). By integrating phenotypic analysis results and the genome data, we expect that more details on the mechanism of ethanol tolerance of *E. coli *cells will be clarified in future studies.

## Methods

### Strain and culture conditions in the evolution experiments

*E. coli *strain W3110 was used as the wild-type strain in this study. The W3110 strain was obtained from National BioResource Project (National Institute of Genetics, Japan). In the evolution experiments, the cells were cultured in 10 mL of M9 minimal medium (2.0 mM MgSO_4_·7H_2_O, 0.1 mM CaCl_2_, 0.5 g/L NaCl, 3.0 g/L KH_2_PO_4_, 17.1 g/L NaHPO_4_·12H_2_O, 1.0 g/L NH_4_Cl, 4.0 g/L glucose; pH 7.0) [40] with or without 5% (v/v) ethanol at the final concentration. Cell culture was performed at 30 °C with shaking at 150 strokes min^-1 ^using water bath shakers (Personal-11, Taitec Co., Saitama, Japan). We diluted the cells into a fresh medium every 24 hours. The cells were maintained in the exponential growth phase by adjusting the initial cell concentration of each dilution to a final cell concentration of less than 0.05 as measured by optical density at 600 nm (OD_600_). The specific growth rate was calculated based on the initial and final cell concentrations of the daily dilution. We confirmed that this calculation of the specific growth rate using 2 time points was accurate (the average absolute deviation is less than 3%) by measuring the specific growth rates of strain P and the evolved strains using OD_600 _values of more than 5 time points. In all evolution experiments, the cells were grown under microaerobic conditions in test tubes with screw cap. The cells after the evolution experiments were stored as glycerol stocks at -80 °C and used for further analysis.

### Phenotype assays of evolved strains

The evolved *E. coli *strains, strain P (parent strain) and the wild-type strain W3110 were inoculated from the glycerol stock to M9 medium and cultured for the preculture. After 8 or 9 generations, cells were inoculated in 10 mL of M9 medium with varying ethanol concentrations (0, 5, 6, 6.5, and 7%). The other conditions were identical to those in the evolution experiments. The experiments in cultures with varying ethanol concentrations were performed 3 times independently. For the evaluation of amino acids and iron ion supplementation, cell growth was analyzed using a biophotorecorder (Toyo Rikakikai CO., Ltd, Tokyo, Japan) with 5 mL of M9 medium with or without ethanol. The supplementation of tryptophan, histidine, valine, leucine, isoleucine were investigated in the final concentrations ranging from 0.1 mM to 10 mM, and the effect of iron ion supplementation was evaluated by adding FeSO_4 _into the medium in the final concentration ranging 1 μM to 4 μM.

### Microarray experiments

For transcriptome analysis, a custom-designed tilling microarray of *E. coli *W3110 in Affymetrix platform was used, which contains approximately 1.5 million perfect-match 21-bp probes for the *E. coli *genome and the corresponding approximately 4.5 million single-base mismatch probes [Ono *et al., *manuscript in preparation]. For the sample preparation, each strain was inoculated from the frozen stock into 10 mL of M9 medium for the preculture. Five-microliter aliquots of the preculture medium cells were inoculated into 10 mL of M9 medium without or with 5% (v/v) ethanol and cultured for 5 generations (without ethanol) or 10 generations (with ethanol). The cells in the exponential growth phase were harvested by centrifugation and stored at -80 °C before RNA extraction. Total RNA was isolated and purified from cells using an RNeasy mini kit with on-column DNA digestion (Qiagen, Hilden, Germany). Synthesis of cDNA, fragmentation and end-terminus biotin labeling were carried out in accordance with the Affymetrix protocols. Hybridization, washing, staining, and scanning were carried out according to the Expression Analysis Technical Manual (provided by Affymetrix). We used the same equipments as shown in a previous study [41].

### Data analysis

To obtain the absolute expression levels of genes from microarray raw data, we used the Finite Hybridization model [17] with slight modifications. We screened expressed genes whose probe signal intensities were significantly higher than the corresponding background signal intensities estimated by a physiochemical model of hybridization [Furusawa *et al., *in preparation]. For the subsequent analysis, we used only genes that were expressed in all samples we inspected (2,317 of a total of 4,499 genes). The expression levels of these genes were normalized using the quantile normalization method [42]. For PCA, The R package was used [43]. The functions of gene products were classified using Gene Ontology Annotation [44]. Information on gene regulation was obtained from RegulonDB [45]. To screen functional categories in which orthologous gene sets with different expression levels are significantly overrepresented, we used a hypergeometric distribution with the following formula:

$$P(X=x|N,M,n)=\frac{\left(\begin{array}{c} M\\ x \end{array}\right)\left(\begin{array}{c} N-M\\ n-x \end{array}\right)}{\left(\begin{array}{c} N\\ n \end{array}\right)}$$

where *N *is the total number of genes inspected, *M *is the number of genes related to a functional category (referred to as "A" below) in the total genes, *n *is the number of genes with the top 5% highest or lowest loading factors in PCA. This probability function describes the probability that we found *x *genes related to category A when we sampled *n *genes having the top 5% highest loading factors. By using this probability function, we obtain the probability *p *that we found more than *k *genes related to category A in the top 5% highest loading factors, as follows:

$$p=\sum_{i=k}^{n}\frac{\left(\begin{array}{l} M\\ i \end{array}\right)\left(\begin{array}{l} N-M\\ n-i \end{array}\right)}{\left(\begin{array}{l} N\\ n \end{array}\right)}$$

Thus, the observation that this *p *value is small enough in real data indicates that genes related to functional category A is significantly overrepresented in the genes having the top 5% highest or lowest loading factors.

## Authors' contributions

THo and KT performed the long-term evolution experiment. THo and SS performed microarray analyses. THo, NO, and CF analyzed the microarray data. THo performed the amino acids supplementation assays. THi and TY participated in the design of the study. THo and CF wrote the manuscript. THi., SS, NO, TY, and H S modified the manuscript. CF and HS conceived the project. All authors read and approved the final manuscript.

## Supplementary Material

Additional file 1 **Supplementary Table 1 - Gene expression data of 14 profiles**. The microarray data after quantile normalization are presented. The value of "0" indicates data which could not be quantified due to its low expression level. Click here for file

Additional file 2 **Supplementary Figure S1 - Changes in expression levels of genes in the parent and tolerant strains**. The log_10_-transformed expression levels of (a) *gut *genes; (b) phosphate transport genes; (c) *manXYZ*; (d) histidine biosynthesis genes; (e) arginine biosynthesis genes; (f) flagellum-related genes; (g) tryptophan biosynthesis genes; (h) branched-chain family amino acid biosynthesis genes; (i) lipopolysaccharide biosynthesis genes; and (j) methionine biosynthesis genes in strain P and tolerant strains A-F with and without ethanol stress are shown. Asterisks indicate genes whose expression levels could not be quantified due to low signal intensities. Click here for file

Additional file 3 **Supplementary Figure S2 - Expression levels of genes regulated by NrdR and OxyR**. The log_10_-transformed expression levels of genes regulated by (a) NrdR and (b) OxyR in strain P (0% or 5% ethanol) and tolerant strains A-F (5%) are presented. The expression levels are sorted in the decreasing order of the expression levels in strain P without ethanol stress. The black dot lines represent the expression levels in strain P without ethanol stress for the reference. A list of genes regulated by NrdR and OxyR in the same order is presented in Additional File 4. Click here for file

Additional file 4 **Supplementary Table 2 - Gene expression data of Fur, NrdR, OxyR regulon members**. The expression data of Fur, NrdR, OxyR regulon members are presented in the same order as shown in Figure 3 and Fig. S2. Click here for file
