# Supplementary figures and images for: Transcriptome analysis of parallel-evolved Escherichia coli strains under ethanol stress
Source: BMC Genomics. 2010 Oct 19;11:579. doi: 10.1186/1471-2164-11-579 (PMC3091726; doi:10.1186/1471-2164-11-579)

Fig. S1(a)

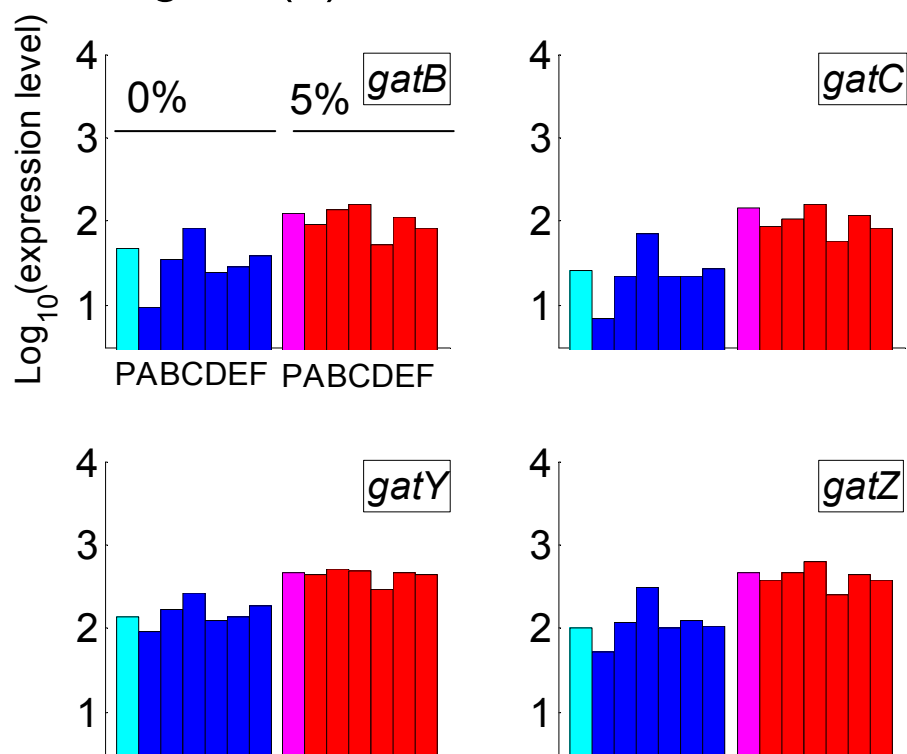

Fig. S1(b)

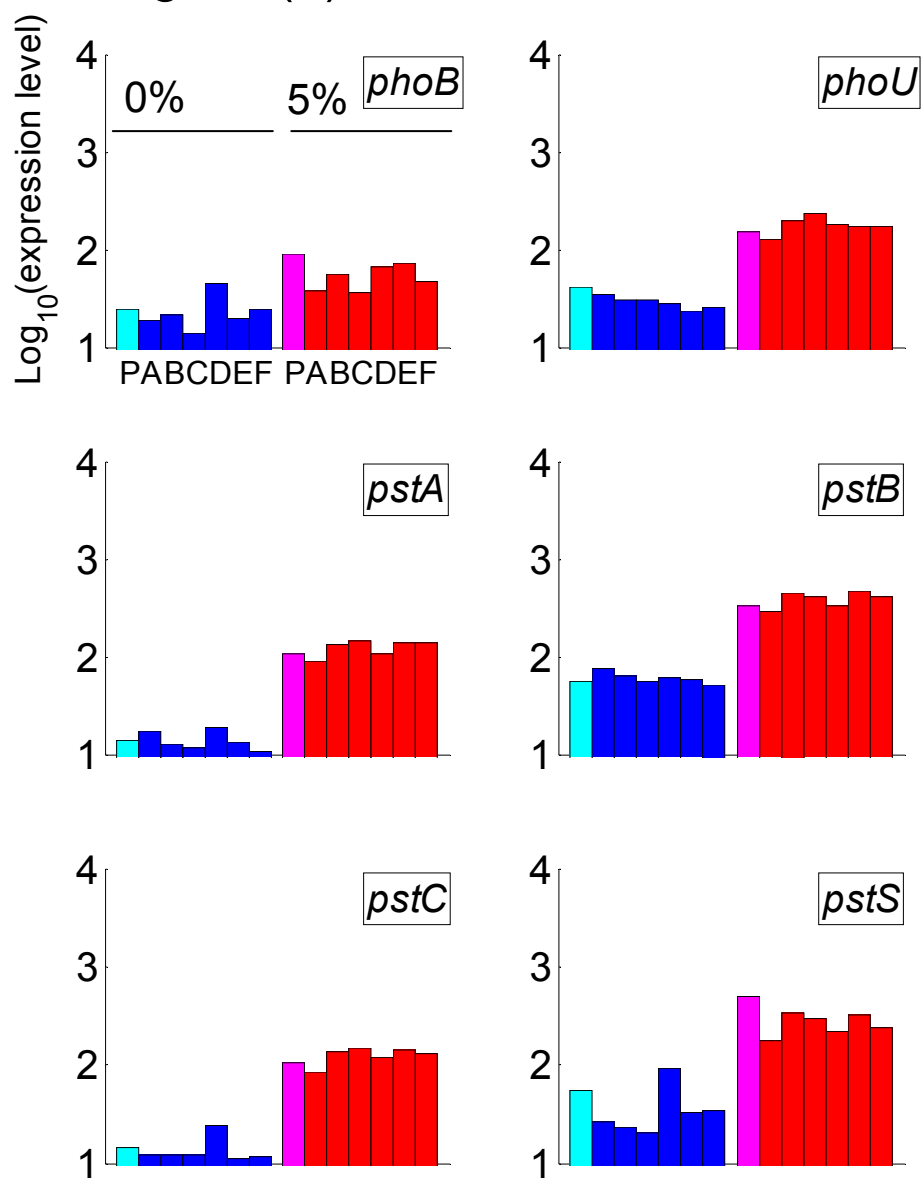

Fig. S1(c)

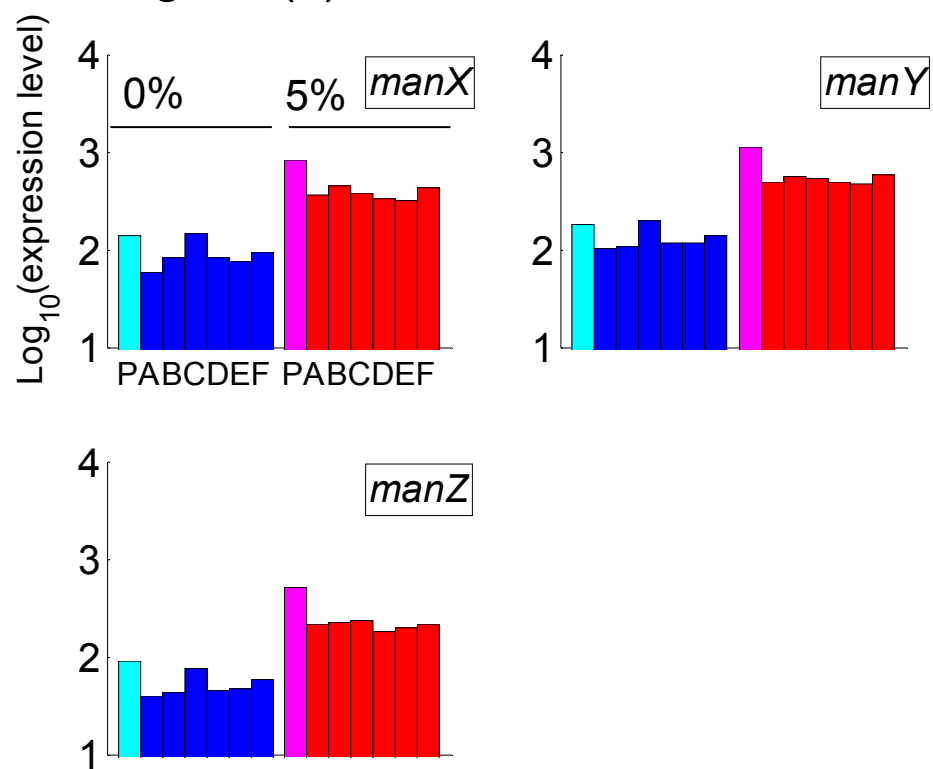

Fig. S1(d)

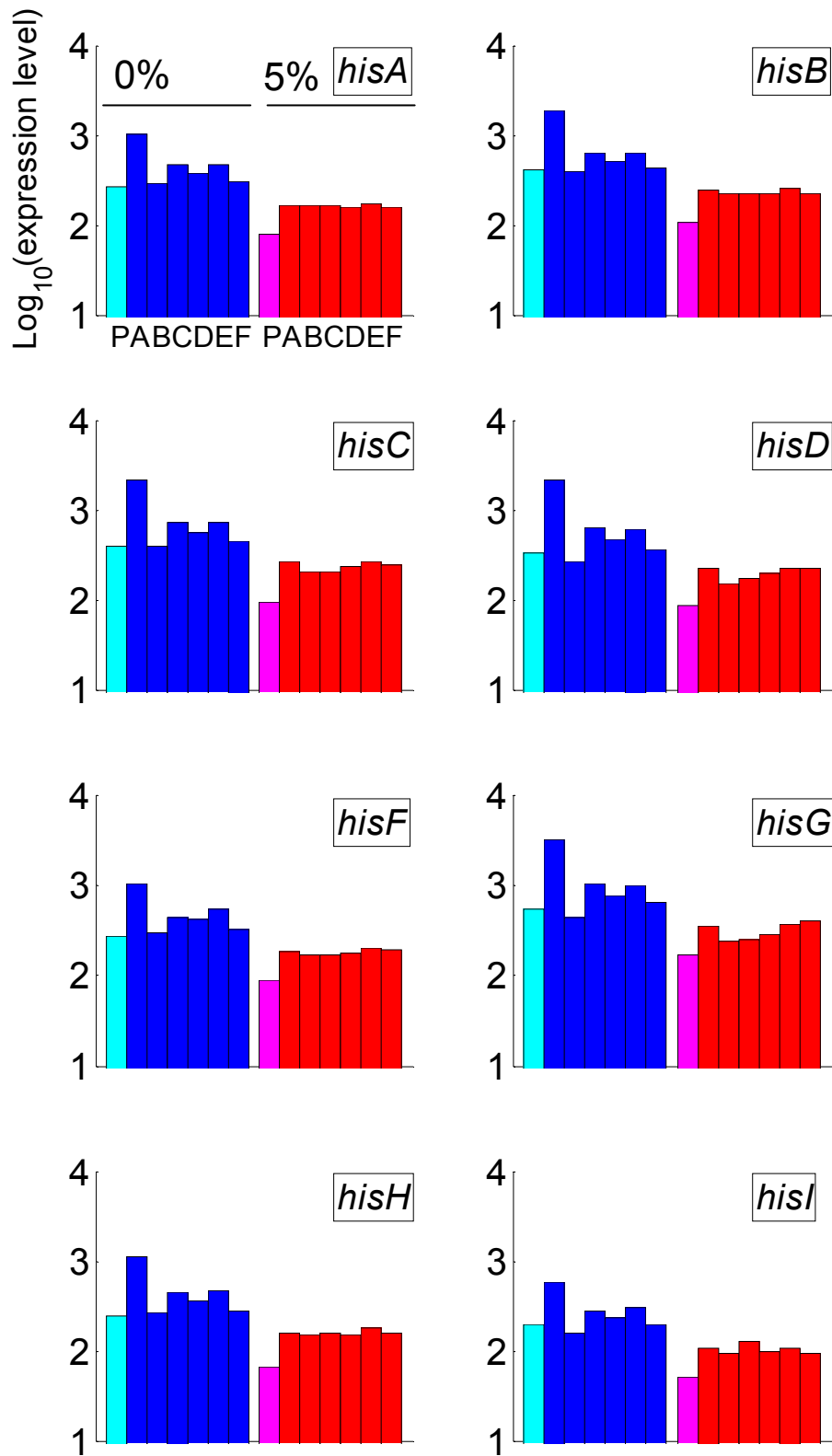

Fig. S1(e)

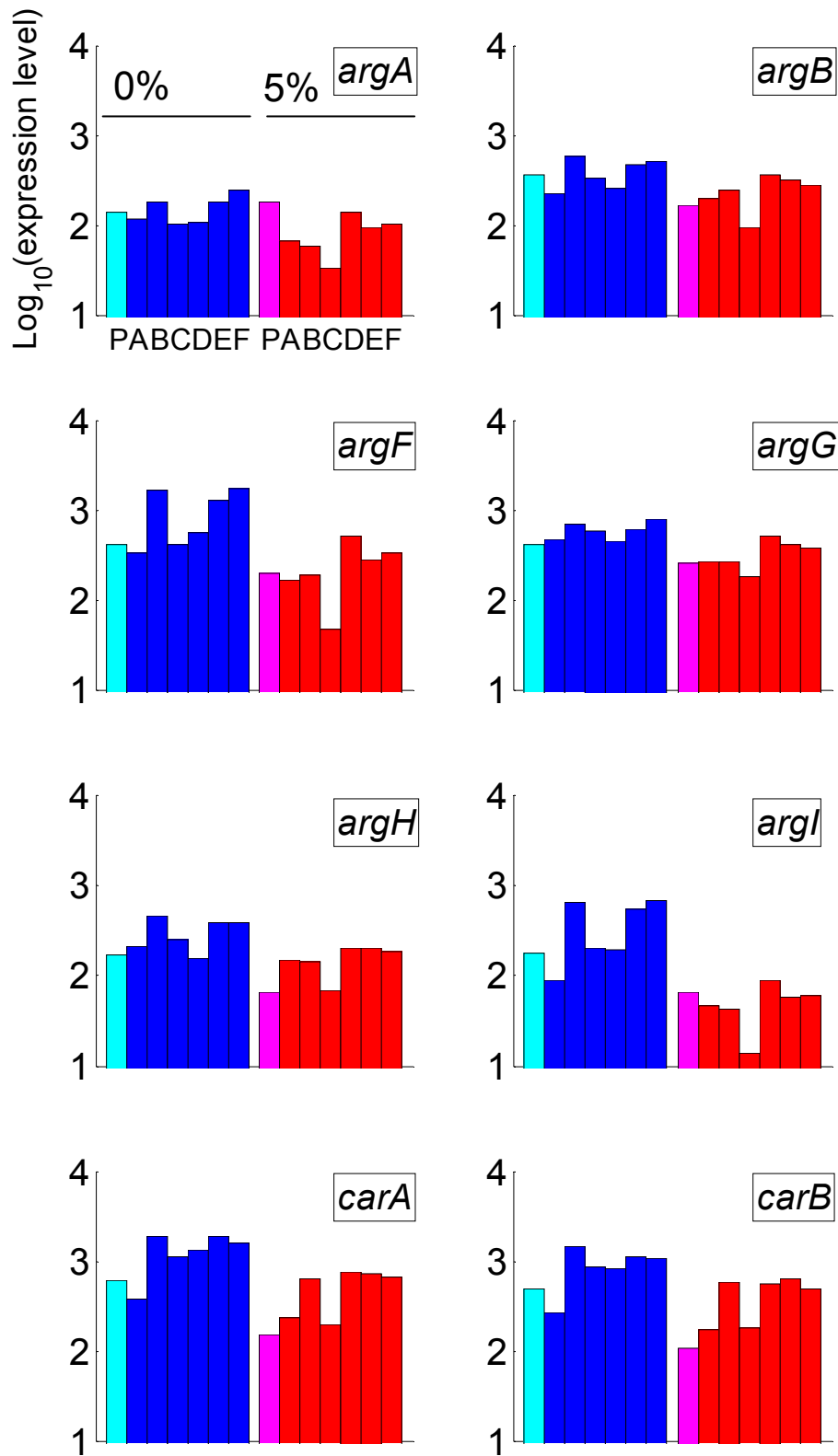

Fig. S1(f)

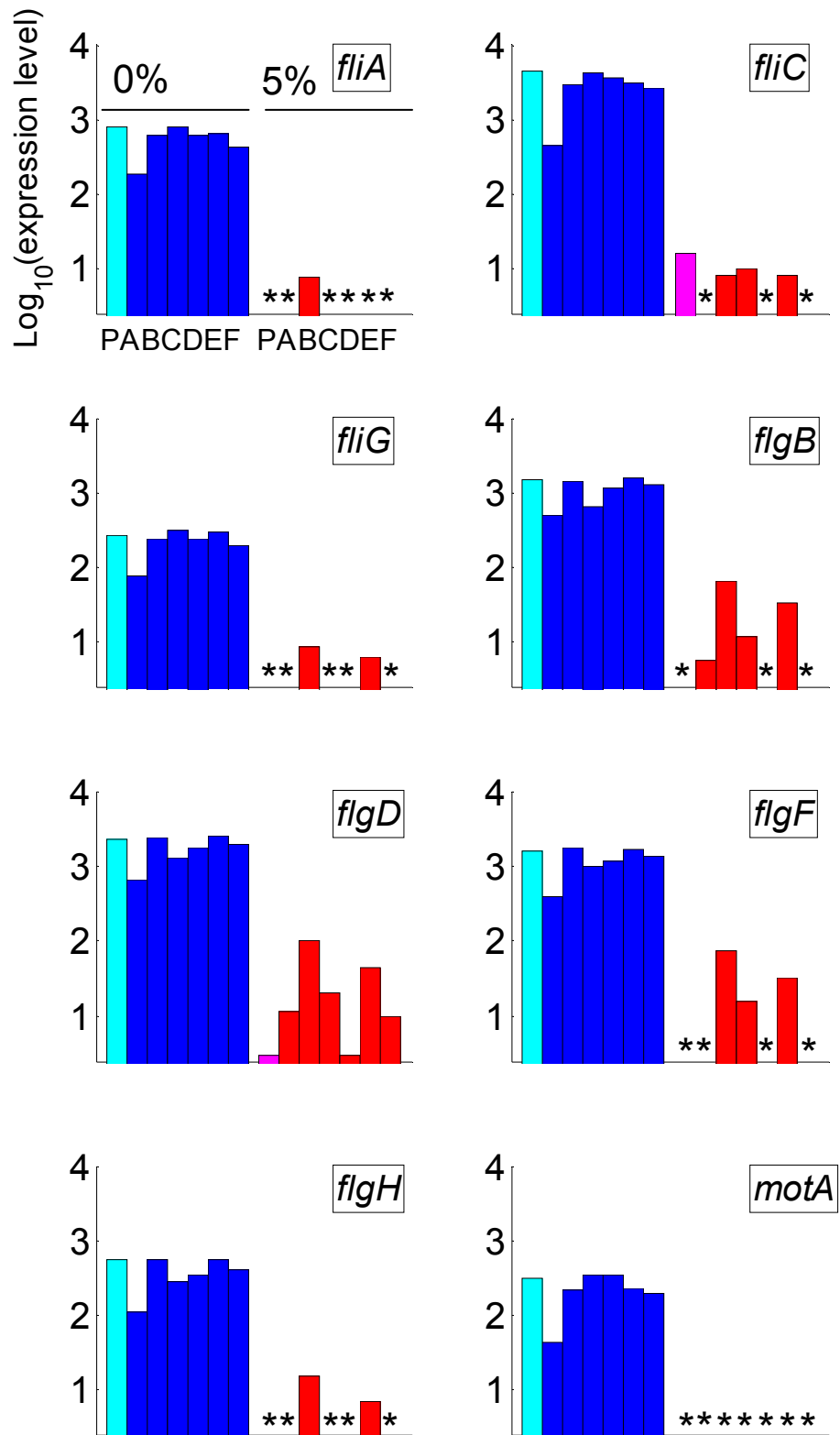

Fig. S1(g)

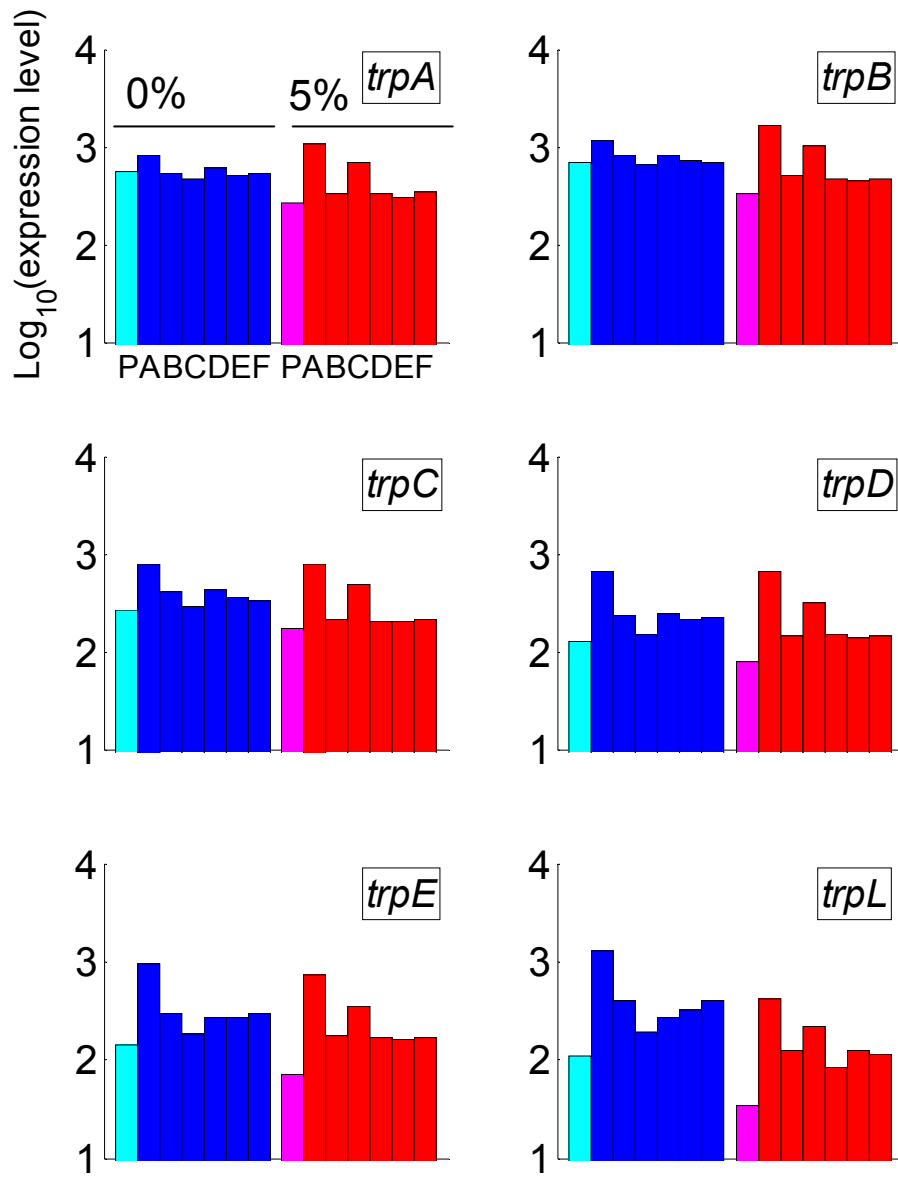

Fig. S1(h)

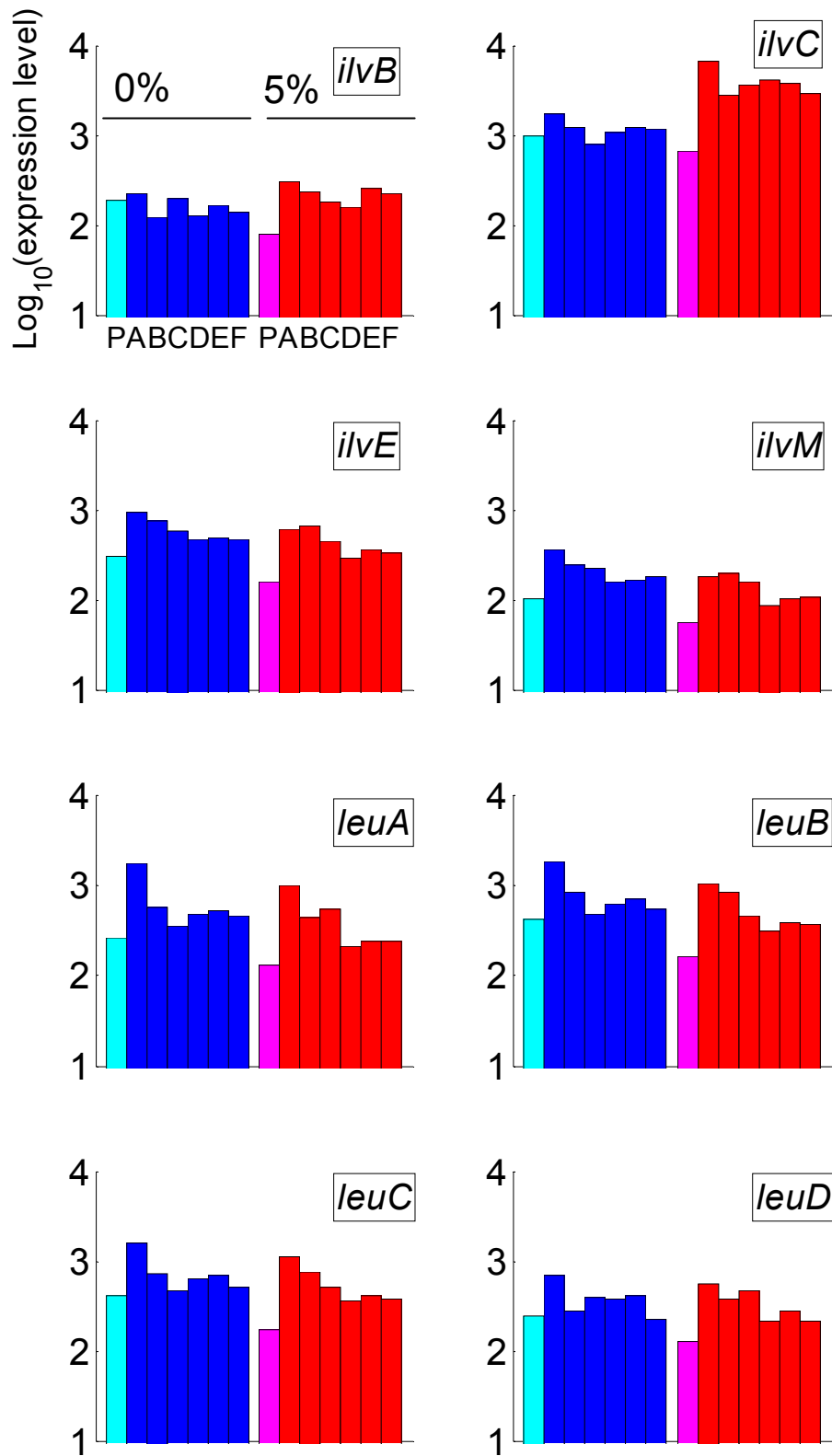

Fig. S1(i)

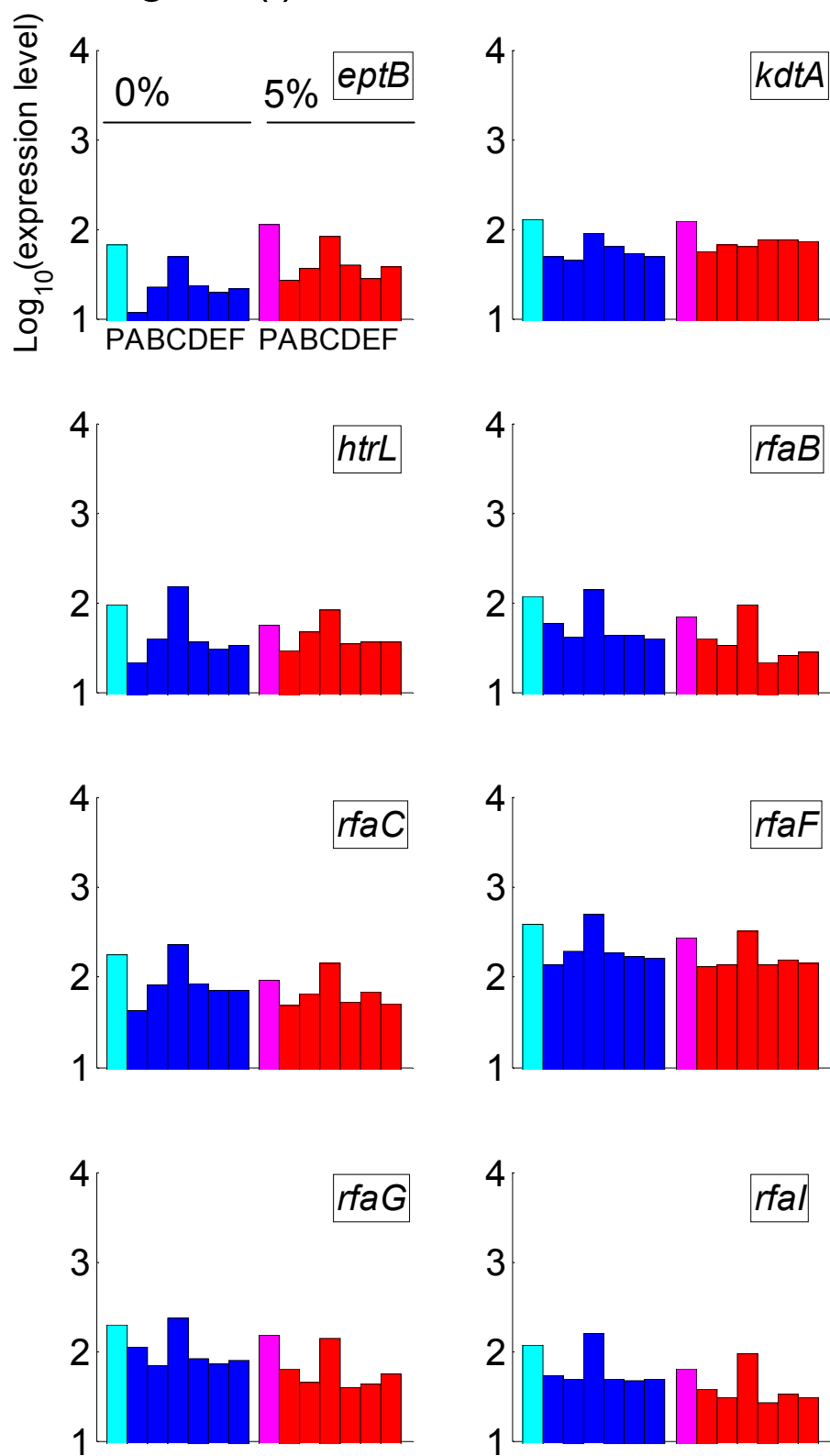

Fig. S1(j)

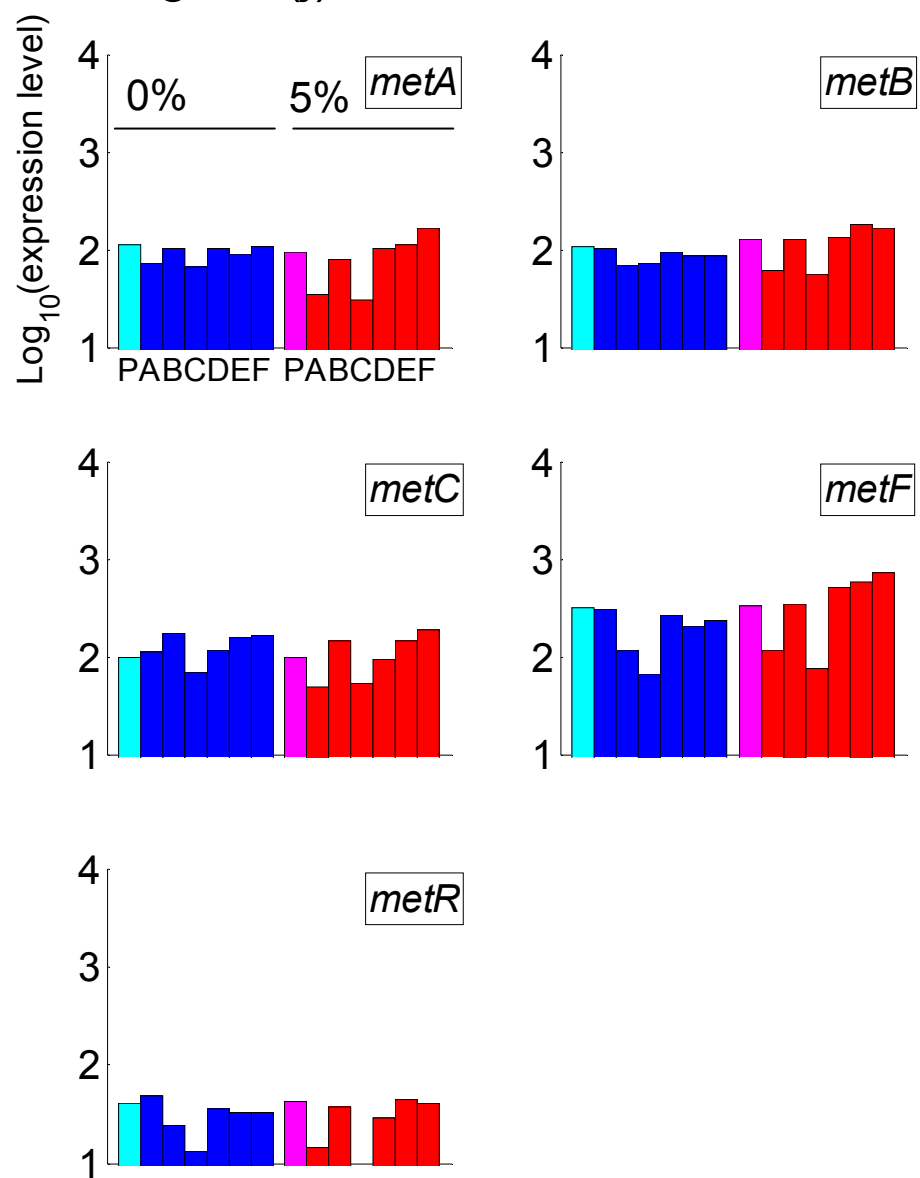

Supplement: Additional file 2 — Supplementary Figure S1 - Changes in expression levels of genes in the parent and tolerant strains. The log10-transformed expression levels of (a) gut genes; (b) phosphate transport genes; (c) manXYZ; (d) histidine biosynthesis genes; (e) arginine biosynthesis genes; (f) flagellum-related genes; (g) tryptophan biosynthesis genes; (h) branched-chain family amino acid biosynthesis genes; (i) lipopolysaccharide biosynthesis genes; and (j) methionine biosynthesis genes in strain P and tolerant strains A-F with and without ethanol stress are shown. Asterisks indicate genes whose expression levels could not be quantified due to low signal intensities. [file 1471-2164-11-579-S2.PDF]

Fig. S2(a)

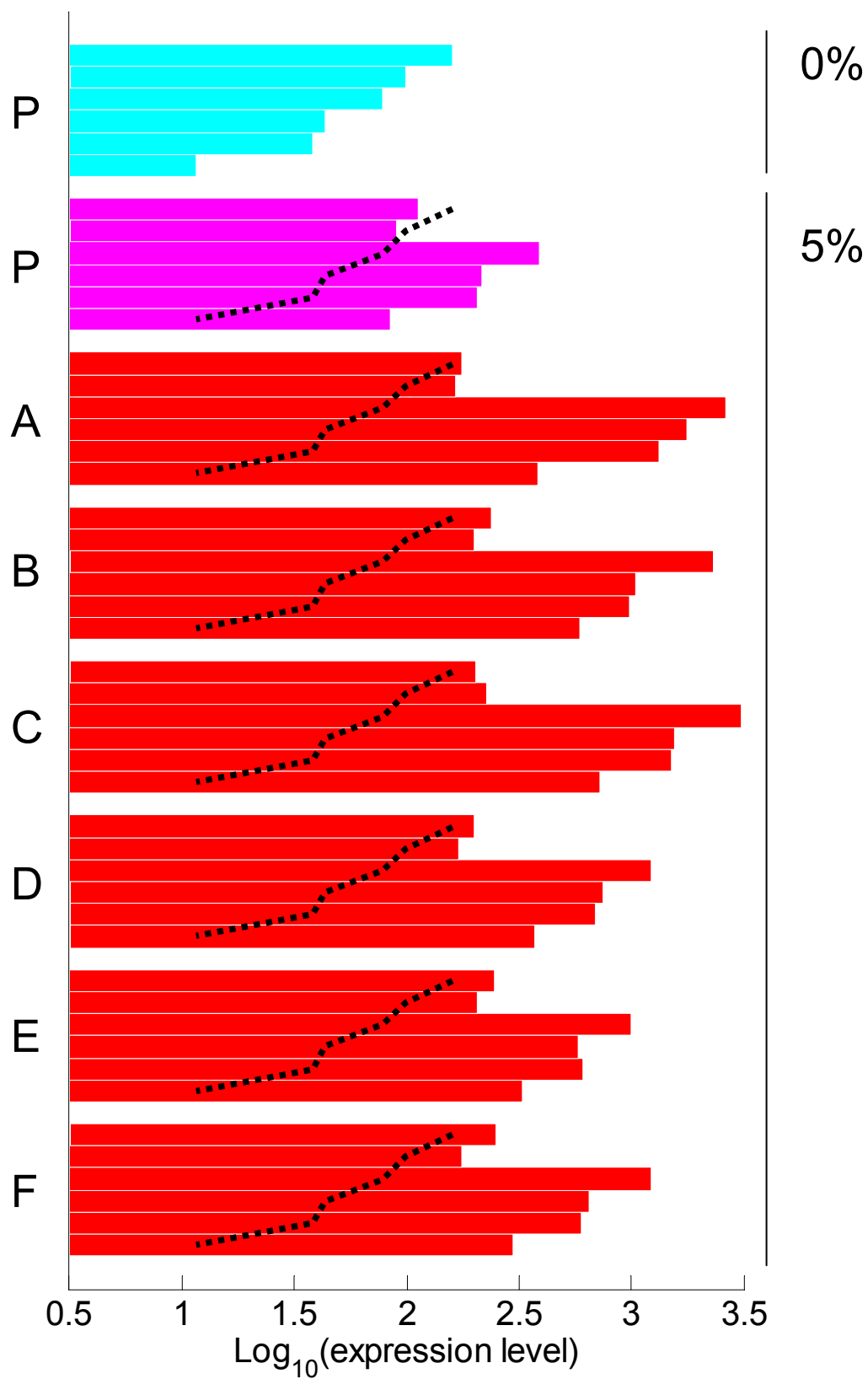

Fig. S2(b)

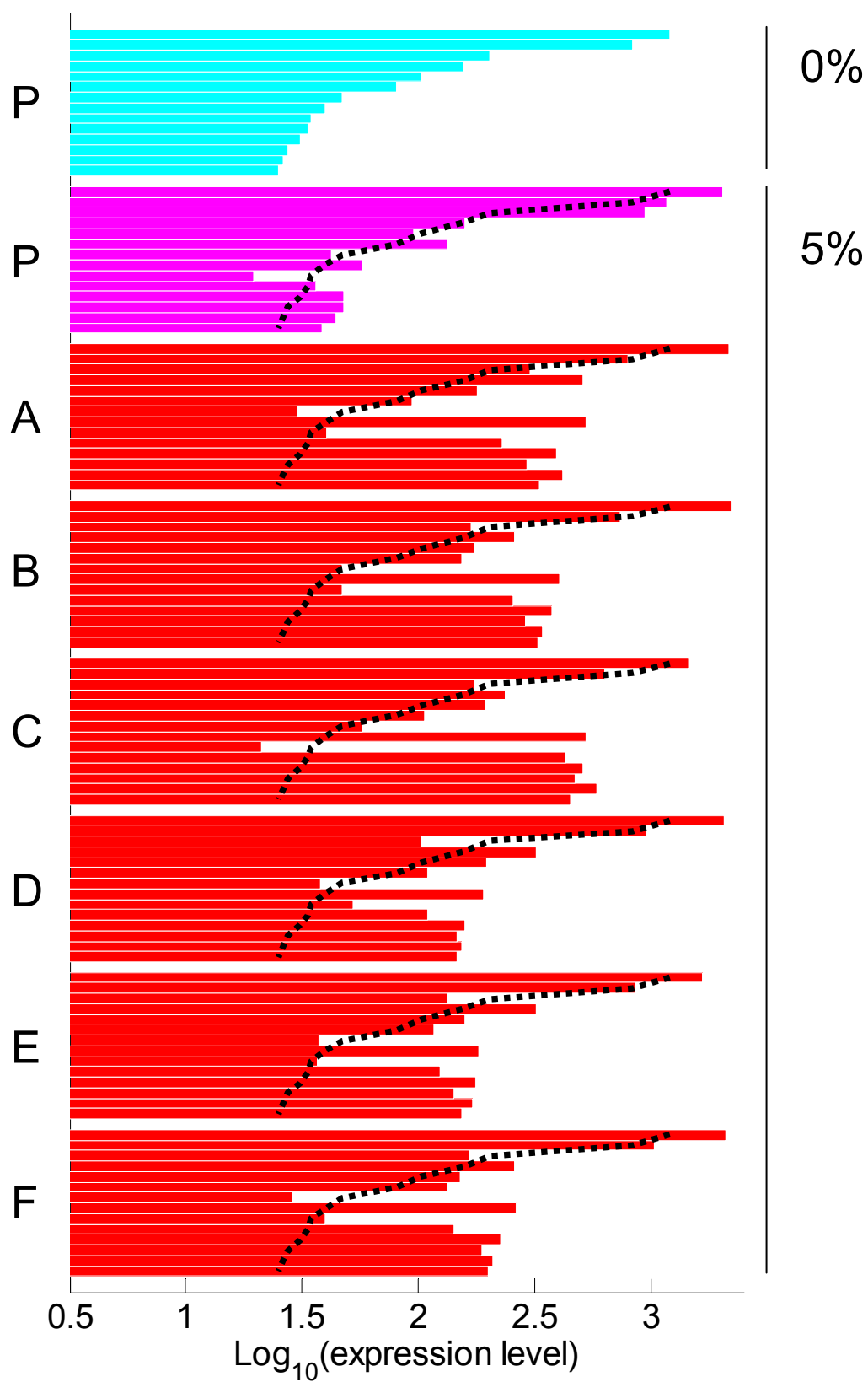

Supplement: Additional file 3 — Supplementary Figure S2 - Expression levels of genes regulated by NrdR and OxyR. The log10-transformed expression levels of genes regulated by (a) NrdR and (b) OxyR in strain P (0% or 5% ethanol) and tolerant strains A-F (5%) are presented. The expression levels are sorted in the decreasing order of the expression levels in strain P without ethanol stress. The black dot lines represent the expression levels in strain P without ethanol stress for the reference. A list of genes regulated by NrdR and OxyR in the same order is presented in Additional File 4. [file 1471-2164-11-579-S3.PDF]
